# Bioprosthetic valve dysfunction and failure after TAVI in bicuspid aortic valve stenosis during one-year follow-up according to VARC-3

**DOI:** 10.1007/s00392-022-02052-9

**Published:** 2022-06-29

**Authors:** Verena Veulemans, Philippe Nuyens, Shouheng Goh, Oliver Maier, Stephan Binnebößel, Jacqueline Heermann, Christian Jung, Ralf Westenfeld, Malte Kelm, Ole de Backer, Tobias Zeus

**Affiliations:** 1 Division of Cardiology, Pulmonology and Vascular Medicine, Heinrich Heine University, Medical Faculty, Moorenstr. 5, 40225 Düsseldorf, Germany; 2 Rigshospitalet, Copenhagen University Hospital, Copenhagen, Denmark; 3 CARID (Cardiovascular Research Institute Düsseldorf), Moorenstr. 5, 40225 Düsseldorf, Germany

**Keywords:** TAVI, Bioprosthetic valve dysfunction, Bicuspid valves, Elderly, Complication, Device success

## Abstract

**Background:**

Transcatheter aortic valve implantation (TAVI) in bicuspid aortic valve (BAV) stenosis has become more frequent in the last years. This may pose challenges for long-time valve durability. Therefore, we aimed to evaluate the prevalence of bioprosthetic valve dysfunction (BVD) with the newest-generation devices in BAV stenosis up to one-year follow-up (FU).

**Methods:**

The primary endpoint was defined as the prevalence of BVD during the first procedural year according to Valve Academic Research Consortium (VARC)-3 criteria. Secondary endpoints were defined as failure in device success and clinical endpoints according to VARC-3.

**Results:**

A total of 107 patients were included. Of these, 34 subjects (31.8%) met the criteria for BVD during a mean FU of 263 ± 180 days, of which 20.2% were already documented after thirty days. Device success after one year was lower in the + BVD cohort (57.6% vs. 98.7%, *p* < 0.0001*). The rates of structural valve deterioration were 6.5%, non-structural valve deterioration (NSVD) 17.8%, subclinical leaflet thickening 10.3%, and endocarditis 0.9%. NSVD was foremost triggered by patient prosthesis mismatch in balloon-expandable valves. Hemodynamic valve deterioration stage 1 and 2 was confirmed in 16.8% of + BVD patients, while stage 1 and 3 bioprosthetic valve failure occurred in 1.9%. There was no impact of BVD on mortality.

**Conclusion:**

There is critical evidence of early BVD after TAVI in BAV during one-year FU in one-third of patients, also lowering device success. The most frequently observed bioprosthetic valve dysfunction was NSVD due to patient prosthesis mismatch following TAVI with a balloon-expandable valve.

**Supplementary Information:**

The online version contains supplementary material available at 10.1007/s00392-022-02052-9.

## Introduction

Transcatheter aortic valve implantation (TAVI) as treatment of aortic stenosis in bicuspid aortic valve (BAV) has become more frequent in the last years. The specific anatomic configurations of BAV pose challenges toward valve performance, outcome, and long-time durability, especially when treating patients with longer life-expectancy [1]. Recent data have shown that newer-generation devices offer similar device success in selected bicuspid compared to tricuspid aortic valves [2, 3], although little is known about prevalence and possible predictors of bioprosthetic valve dysfunction (BVD) during follow-up. Recently, the Valve Academic Research Consortium 3 endpoint definitions for aortic valve clinical research [4] were released with critical updates concerning an adequate interpretation of BVD. Structured data on the prevalence and an in-depth analysis of clinical, anatomical, and technical risk factors for BVD are lacking. Therefore, we aimed to evaluate the prevalence of BVD during one-year follow-up in patients who underwent TAVI in BAV stenosis.

## Methods

### Study population

Consecutive patients with symptomatic bicuspid severe aortic stenosis (AS) who underwent either transfemoral (TF), transapical (TA) or alternative access TAVI with newer-generation devices (SAPIEN 3, Edwards Lifesciences; Evolut R/Pro platform, Medtronic) were included in this retrospective analysis. Valve-in-valve procedures, pure aortic regurgitation, TAVI with other bioprosthetic valves, and procedures without appropriate MSCT data were excluded. All procedures were performed according to current guideline recommendations and under local or general anesthesia.

All patients provided written informed consent for TAVI and for the use of their clinical, procedural, and follow-up data in research. The study procedures were conducted in accordance with the Declaration of Helsinki, and the institutional Ethics Committee of Heinrich-Heine University approved the study protocol (4080).

### Study endpoints

The primary endpoint was defined as the prevalence of BVD during one-year follow-up (FU) according to Valve Academic Research Consortium (VARC)-3 criteria [4]. Secondary endpoints were defined as failure in device success and clinical endpoints according to VARC-3.

### Procedural details and 3D image analysis of MSCT

MSCT images were transferred to a dedicated workstation for 3-dimensional (3D) volume-rendered reconstruction (3 mensio Structural Heart™, Pie Medical Imaging BV, Maastricht, The Netherlands) after the procedure according to TAVI-related standardized recommendations for CT image acquisition [5]. Dimensions were determined with the use of workstation tools. Tubular (tube), flared or tapered configurations of the aortic root were identified from the relation of the annulus to the inter-commissural distance (ICD) according to the BAVARD-registry [6]. The total aortic valve calcification (AVC) and calcium amount of the upper LVOT expressed as recalculated Agatston Units (AU) adapted from the calcium volume was calculated. All MSCT-reconstructions, echocardiographic interpretations, and analyses were done by experienced level 3 readers. In a subset of patients (23%), post-procedural MSCT was performed. The implantation strategy was retrospectively analyzed according to the CASPAR algorithm [7] as described before.

### VARC-3 definitions of bioprosthetic valve dysfunction (BVD) (4)

For the details, please see **Supplementary material – Methods.**

### Statistical analysis

The collected data included patient characteristics, imaging findings, periprocedural in-hospital data, laboratory results and follow-up data. Continuous data are described by the mean and standard deviation, and categorical variables by frequencies and percentages. Continuous variables were compared using Student’s t-test, categorical variables were compared using Fisher’s exact test. The data analysis was performed using the statistical software SPSS (version 27.0.1, SPSS Inc., Chicago, IL, USA), GraphPad Prism (version 6.0, GraphPad Software, San Diego, CA, USA), and Wizard 2- Statistics & Analysis (Evan Miller). All statistical tests were 2-tailed, and a value of p < 0.05 was considered statistically significant.

## Results

From 2016 to December 2020, in total 109 patients were identified at the Heart Centre Düsseldorf in Germany or at Rigshospitalet in Copenhagen, Denmark. Since one patient had died (early mortality) and one patient was immediately converted to surgery, we finally included 107 patients in the analyses. Follow-up (FU) was completed 3 months to 1-year FU in this. The study cohort was further separated into patients without and with detection of BVD during a mean FU of 263 ± 180 days (-BVD: n = 73; 68.2%; + BVD: *n* = 34; 31.8%).

### Baseline characteristics

Baseline characteristics did differ according to the particular risk profile of patients without and with BVD. For example, patients with BVD had a lower left ventricular function (LVEF; -BVD vs. + BVD: 54.0 ± 10.9 vs. 48.4 ± 13.5; *p* = 0.023*) and smaller aortic valve area (AVA; -BVD vs. + BVD: 0.75 ± 0.2 vs. 0.64 ± 0.2; *p* = 0.007*). All other dimensions of the aortic root as well as calcification distribution were similar (Supplementary material – Table S1).

### General procedural characteristics

Procedural details and clinical outcomes are displayed in Table 1. Implantation based on annular sizing was realized in more than two-thirds of cases without differences between the cohorts. Implantation strategy according to the CASPAR algorithm was lower in the + BVD cohort (48.9%) but statistical similar compared to the non-BVD cohort (56.9%). Post-dilatation was more frequently observed in BVD patients (-BVD vs. + BVD: 17.3% vs. 38.2%; *p* = 0.018*).

**Table 1:** Procedural Characteristics

Procedural data	Overall (*n* = 109; 100%)	No BVD (*n* = 75; 68.8%)	BVD (*n* = 34; 31.2%)	*p* value
TF access	106 (97.3)	73 (97.3)	33 (97.1)	0.935
TA access	2 (1.8)	2 (2.7)	0 (0.0)	0.337
Other access	1 (0.9)	0 (0.0)	1 (2.9)	0.136
EDWARDS Sapien 3™	35 (32.1)	23 (30.7)	12 (35.3)	0.632
23 mm	2 (5.71)	2 (8.7)	0 (0.0)	0.293
26 mm	10 (28.6)	6 (26.2)	4 (33.3)	0.652
29 mm	23 (65.7)	15 (65.2)	8 (66.7)	0.932
Evolut R™	54 (49.5)	40 (53.3)	14 (41.2)	0.240
23 mm	1 (1.9)	1 (2.5)	0 (0.0)	0.550
26 mm	13 (24.1)	11 (27.5)	2 (14.3)	0.320
29 mm	16 (29.6)	12 (30.0)	4 (28.6)	0.920
34 mm	24 (44.4)	16 (40.0)	8 (57.1)	0.267
Evolut Pro™	20 (18.4)	12 (16.0)	8 (23.5)	0.347
26 mm	5 (25.0)	4 (33.3)	1 (12.5)	0.292
29 mm	15 (75.0)	8 (66.7)	7 (87.5)	0.292
Implantation based on annular sizing	92 (84.4)	62 (82.7)	30 (88.2)	0.458
Implantation according to CASPAR	57 (54.3)	41 (56.9)	16 (48.9)	0.419
Contrast, ml	104.2 ± 35.2	100.3 ± 35.9	112.7 ± 39.0	0.089
Fluoroscopy time, min	23.0 ± 8.3	22.2 ± 8.1	24.8 ± 8.5	0.139
Dose Area Product, Gy x cm^2^	1.960 [950–3.772]	2.025 [1.070–3.704]	1.516 [918–4.180]	0.678
Pre-dilatation	88 (80.7)	59 (78.7)	29 (85.3)	0.416
Post-dilatation	26 (23.9)	13 (17.3)	13 (38.2)	0.018*
AR ≥ II°	4 (3.8)	1 (1.3)	3 (8.8)	0.054
Intraprocedural complications				
Immediate stroke	1 (0.9)	0 (0)	1 (2.9)	0.136
Aortic dissection	0 (0)	0 (0)	0 (0)	1.000
Annulus rupture	0 (0)	0 (0)	0 (0)	1.000
Coronary obstruction	2 (1.83)	0 (0)	2 (5.88)	0.034*
Vascular complications	10 (9.2)	7 (9.3)	3 (8.8)	0.932
Valve dislocation	0 (0)	0 (0)	0 (0)	1.000
Conversion to surgery	1 (0.9)	1 (1.3)	0 (0)	0.499
Need of 2nd valve	0 (0)	0 (0)	0 (0)	1.000
Tamponade	1 (0.9)	1 (1.3)	0 (0)	0.499
CPR	1 (0.9)	0 (0)	1 (2.9)	0.136
Immediate Procedural death	0 (0.0)	0 (0.0)	0 (0.0)	1.000

Values are mean ± SD, median ± interquartile range or *n* (%)

*AR* aortic regurgitation, *CPR* cardiopulmonary resuscitation, *TA* transapical, *TF* transfemoral

*p value < 0.05

### Thirty-day outcome and functional status

Table 2 summarizes the main clinical and echocardiographic outcomes observed at 30 days according to the recently released VARC-3 criteria. In general, the clinical outcomes were similar between both groups except from a higher number of cerebrovascular events in + BVD patients (CVE; -BVD vs. + BVD: 0.0% vs. 5.9%; *p* = 0.034*) and to BVD linked adverse events that are listed in Table 2 and of which two-third already were documented during 30-days FU. The primary device success was statistically comparable in both groups but tended to be lower in the + BVD collective (85.3% vs. 96.0%).

**Table 2:** 30-day outcome according to VARC-3

Post-procedural outcome	Overall (*n* = 109; 100%)	No BVD (*n* = 75; 68.8%)	BVD (*n* = 34; 31.2%)	*p* value
30-day mortality	1 (0.9)	1 (1.3)	0 (0)	0.499
Peri- and post-procedural MI	0 (0)	0 (0)	0 (0)	1.000
CVE (NeuroARC 1)	2 (1.83)	0 (0)	2 (5.9)	0.034*
Hemorrhagic stroke	2 (1.83)	0 (0)	2 (5.9)	0.034*
Severity (mRS < 2)	1 (0.9)	0 (0)	1 (2.9)	0.136
Severity (mRS > 2)	1 (0.9)	0 (0)	1 (2.9)	0.136
TIA	0 (0)	0 (0)	0 (0)	1.000
Bleeding	16 (14.7)	13 (17.3)	3 (8.8)	0.245
Type I	7 (6.4)	6 (8.0)	1 (2.9)	0.318
Type 2	6 (5.5)	5 (6.7)	1 (2.9)	0.429
Type 3	3 (2.8)	2 (2.7)	1 (2.9)	0.935
Type 4	0 (0)	0 (0)	0 (0)	1.000
VASC	21 (19.3)	16 (21.3)	5 (14.7)	0.416
Major VASC	6 (5.5)	5 (6.7)	1 (2.9)	0.429
Minor VASC	14 (12.8)	10 (13.3)	4 (11.8)	0.821
Closure Device Failure	6 (5.5)	3 (4.0)	3 (8.8)	0.306
Access-related non-VASC	0 (0)	0 (0)	0 (0)	1.000
AKI I-IV	5 (4.6)	5 (6.7)	0 (0)	0.123
AKI I	2 (1.8)	2 (2.7)	0 (0)	0.337
AKI II	1 (0.9)	1 (1.3)	0 (0)	0.499
AKI III	2 (1.8)	2 (2.7)	0 (0)	0.337
AKI IV (new RRT)	0 (0)	0 (0)	0 (0)	1.000
New AVB (I-III°)	20 (18.4)	16 (21.3)	4 (11.8)	0.232
New LBBB/RBBB	22 (20.2)	12 (16.0)	10 (29.4)	0.213
New PPI	17 (15.6)	13 (17.3)	4 (11.8)	0.458
TAVI-related Reintervention	4 (3.7)	3 (4.0)	1 (2.9)	0.785
Cardiac Structutal Complications	4 (3.7)	2 (2.7)	2 (5.9)	0.263
Minor	3 (2.8)	1 (1.3)	2 (5.9)	0.179
Major	2 (1.8)	1 (1.3)	1 (2.9)	0.337
Functional data				
Hint on BVD	22 (20.2)	0 (0)	22 (64.7)	< 0.001*
SVD	5 (4.6)	0 (0)	5 (14.7)	< 0.001*
NSVD	11 (10.1)	0 (0)	11 (32.4)	< 0.001*
Endocarditis TAVI	0 (0)	0 (0)	0 (0)	1.000
Leafletthrombosis/HALT	7 (6.4)	0 (0)	7 (20.6)	< 0.001*
Vmax (m/s)	2.1 ± 0.5	2.0 ± 0.4	2.2 ± 0.5	0.037*
dPmean (mmHg)	10.0 ± 4.4	9.3 ± 3.9	11.5 ± 5.2	0.013*
EOAi (cm^2^/m^2^)	1.0 ± 0.2	1.1 ± 0.2	0.9 ± 9.2	0.001*
Moderate-to-severe PPM	8 (7.4)	0 (0)	8 (23.5)	< 0.001*
EOAi < 0.85 cm^2^/m^2 (BMI<30)^	8 (11.0)	0 (0)	8 (23.5)	< 0.001*
EOAi < 0.70 cm^2^/m^2 (BMI≥30)^	0 (0)	0 (0)	0 (0)	1.000
PVL ≥ II°	3 (2.8)	0 (0)	3 (8.8)	0.009*
Device success	101 (92.7)	72 (96.0)	29 (85.3)	0.105
Medication at discharge/30-days				
APT mono	14 (13.1)	10 (13.7)	4 (11.8)	0.782
DPT	43 (40.2)	46 (63.0)	18 (52.9)	0.322
OAC mono	19 (17.8)	11 (15.1)	8 (23.5)	0.286
OAC + APT	9 (8.4)	5 (6.9)	4 (11.8)	0.394

Values are mean ± SD, median ± interquartile range or *n* (%)

*AKI* acute kidney injury, *APT* antiplatelet therapy, *AVB* atrioventricular block, *BMI* body mass index, *CVE* cerebrovascular events, *DPT* dual antiplatelet therapy, *EOA*_*i*_ effective orifice area (index), *HVD* Hemodynamic valve deterioration, *LBBB* left-bundle branch block, *NSVD* Non-structural valve deterioration, *OAC* oral anticoagulation, *PPI* permanent pacemaker therapy, *PVL* paravalvular leakage, *RBBB* right-bundle branch block, *RRT* renal replacement therapy,

*p value < 0.05

### One-year outcome and functional status

After one year, two deaths were documented in the -BVD cohort of unknown causes (i.e. cardiovascular death) and one death in the + BVD cohort due to endocarditis/BVF stage 3 (-BVD vs. + BVD: 2.7% vs. 2.9%; *p* = 0.814).

**Fig. 1:**
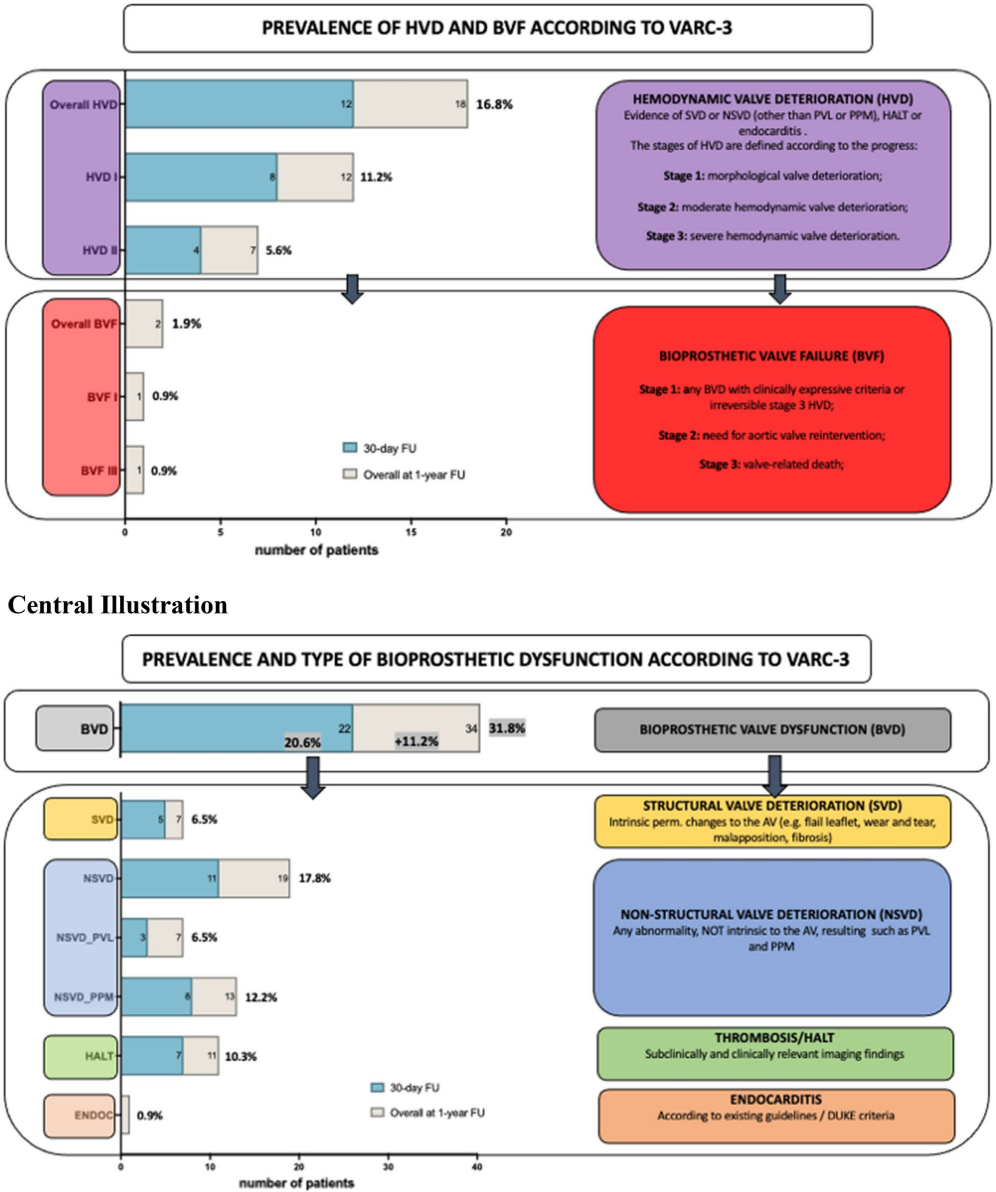
Central Illustration: Prevalence of bioprosthetic Valve Dysfunction (BVD) during 30-day and One-year FU. Prevalence of Hemodynamic Valve Deterioration (HVD) and Bioprosthetic Valve Failure (BVF) during 30-day and One-year FU

SVD was documented in 7 patients (6.5%), NSVD in 19 patients (17.8%), HALT/RELM in 11 patients (10.3%), and endocarditis in one patient (0.9%). In-depth differentiation of BVD is illustrated in the Central Illustration. Moderate PVL was the reason in 6.5% of NSVD, while PPM was documented in 12.2% of NSVD cases. HVD was confirmed in 16.8% of + BVD patients, while BVF occurred in 2 patients (stage 1 and 3; 1.9%; Fig. 1).

**Fig. 2:**
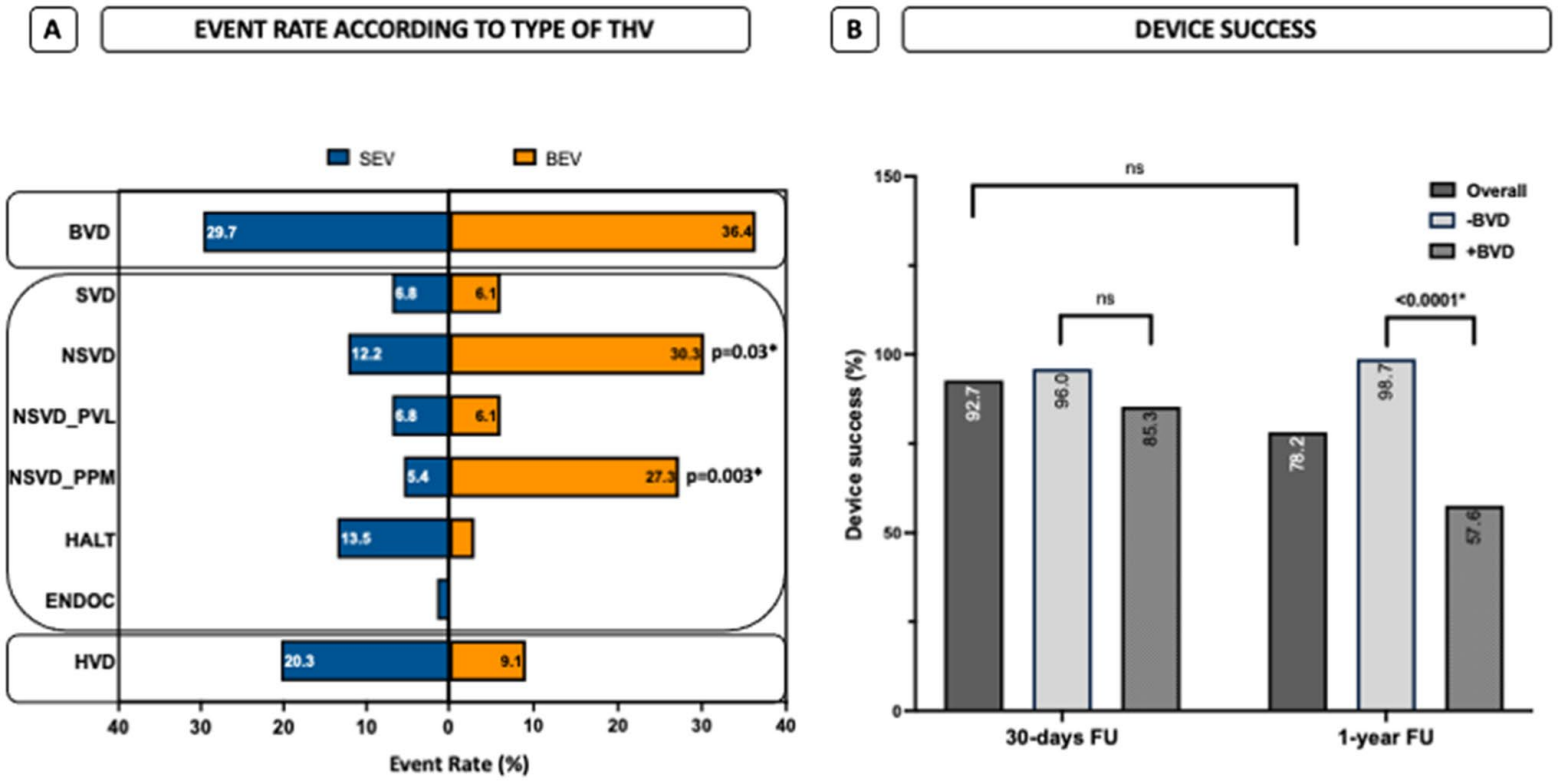
Event rate according to the type of Transcatheter Heart Valve (THV) and Device success. **A** Frequency distribution of BVD in self- (SEV) and balloon-expandable valves (BEV). **B** Device success during time course in -BVD- and + BVD patients

When focusing on a potential impact of the kind of transcatheter heart valve (THV) on BVD event rate, no difference in overall BVD between self-expandable (SEV) and balloon-expandable (BEV) valves, but a higher incidence of overall NSVD (12.2% vs. 30.2%; *p* = 0.03), foremost triggered by PPM (5.4% vs. 27.3%; *p* = 0.003) in BEV, was documented (Fig. 2A).

Compared to the 30-day FU, device success after one year was significant lower in the + BVD collective (57.6% vs. 98.7%, *p* < 0.0001*; Fig. 2B), comparably triggered by SEV and BEV. Concerning functional parameters, the mean valvular gradients were higher by average in the + BVD cohort as compared to -BVD patients (dPmean; -BVD vs. + BVD: 7.9 ± 3.7 vs. 11.6 ± 6.7; *p* = 0.037*, data not shown).

## Discussion

As staging and interpretation of BVD were handled heterogenous in the last years, the recently released VARC-3 definitions now enable a streamlined interpretation algorithm for more homogenous results. To our knowledge, this is the first study addressing VARC-3 definitions for BVD in bicuspid TAVI. The main read-outs of our dual-center retrospective study revealed that:

1. There is evidence of frequently observed BVD after TAVI in bicuspid aortic valves during 30-day (20.2%) and 1-year FU (31.8%), also lowering device success.
2. The most frequently observed component of BVD was NSVD (17.8%), foremost triggered by patient prosthesis mismatch (PPM) in balloon-expandable valves (BEV).
3. There was no impact of BVD on thirty-day or one-year mortality.

Bioprosthetic dysfunction is known to consist of both structural and non-structural alterations that have now been systemized according to etiology and severity, in line with current literature [8, 9]. Thus, four columns of BVD have been classified: SVD, NSVD, valve thrombosis with subclinically or clinically relevant HALT/RELM, and endocarditis. The new VARC-3 criteria now streamlined the current and slightly differing definitions of SVD. They allow for differentiation of the SVD-related new term of hemodynamic valve deterioration (HVD) from PPM-related residually high transprosthetic gradients in the meaning of NSVD to avoid an overestimation of SVD definition based on elevated gradients. Furthermore, in-depth subclassifications of SVD have also been provided, including morphological changes (stage 1) despite elevated gradients. In terms of PPM, the EOAi was thoroughly distinguished, differentiating between an EOAi < 0.85 cm^2^/cm^2^ and EOAi < 0.70 cm^2^/cm^2^ in obese patients to avoid an overestimation of NSVD. In tricuspid valves, the differing interpretation of BVD became already evident in the last years using different criteria for both surgical and transcatheter devices. Par example, the definition of SVD in the VIVID consensus document [9] introduced the concept of progressive SVD stages into the main criteria of the EAPCI/ESC/EACTS definitions [8], also including specific management recommendations according to the degree and kind of dysfunction. Thus, different stages of bioprosthetic valve failure (BVF) were incorporated into VARC-3, allowing to assess clinically relevant consequences of BVD and including the need for valve-related death and re-intervention. These changes might help to better differentiate the underlying conditions and resulting consequences in the management of BVD, also giving in-detail recommendations for an appropriate imaging following bioprosthetic valve implantation.

Considering all possible alterations, BVD showed a high prevalence (31.8%) in bicuspid valves during one-year FU in this study, of which two-thirds of all events were already documented during 30-day FU (20.2%). However, BVD in this study was primarily due to NSVD, foremost triggered by PPM in balloon-expandable valves that are known to lead to PPM in more than fifty percent of tricuspid TAVI [10]. According to VARC-3, PPM was thoroughly adapted to the recommended EOAi differentiating between obese and non-obese patients. In general, TAVI is associated with a lower prevalence of PPM than surgical aortic valve replacement, showing the lowest prevalence using self-expanding valves with a supra-annular design [11]. Regarding the association to longer-term outcomes, a valuable hemodynamic performance across bicuspid patients undergoing TAVI may be crucial for long-term durability [12].

In contrast, SVD was recognized with 6.5% within our study, nearly 5% already during 30-day FU. Until now, there were no data on early SVD stages in bicuspid TAVI. According to current literature, the medium long-term risk of severe SVD seems to be low (up to 2.5%) for 4–8 years FU [13] in tricuspid valves. Thus, the amount of short- and mid-term SVD in this study seems to be comparatively high. Recently, *Forrest *et al*.* [14] showed favorable 30-day results in bicuspid low-risk patients undergoing TAVI with self-expandable valves, counting for significantly elevated gradients in only 1.4% of cases. However, the analysis was almost based on VARC-2 criteria and realized without in-detail BVD analysis. The overall device success with 95% was comparable to this study with 93% after thirty days, which sounds valuable for both studies at first glance. Nevertheless, when differentiating into BVD and non-BVD cases, device success after thirty days and one year was dramatically lower in the + BVD cohort (85% vs. 57% respectively). To reiterate: More than one-third of patients showing any BVD event, formally performed with device failure during the one-year course. A phenomenon that must be considered when widely expanding TAVI in bicuspid low-risk patients.

Subclinical HALT with reduced leaflet motion (RELM, i.e. leaflet restriction) was diagnosed in 10.3% of our cases by imaging findings (MSCT and TEE), approximately 7% already documented during 30-day FU and the majority in self-expandable valves. At this point, it must be pronounced that the evaluation of HALT should be performed by cardiac CT with high spatial resolution and multiplanar reformats. Post-procedural MSCT was only performed in approximately 23% of cases in this study but predominantly in self-expandable devices (70% vs. 30% of all performed post-treatment CTs), supposing a possible under-estimation and selection bias as well, as TAVI in BAV was predominantly performed with self-expandable valves (~ 68% of cases). However, leaflet thrombosis can alternatively be diagnosed by TEE to evaluate leaflet thickness and motion [4], which was the case in 4% of patients due to already visible alterations and abnormal flow conditions recorded by transthoracic echocardiography. Furthermore, the frequent but commonly subclinical phenomenon of HALT, also synonymous described as subclinical leaflet thrombosis, must be distinguished from clinically relevant valve thrombosis. However, the prevalence of HALT in this study is in line with the current reported evidence in bicuspid valves [15]. In general, HALT has been described in 5–40% of all patients who undergo post-procedural MSCT [16] and is known to be probably associated with composites of strokes or TIA when becoming clinically relevant. As a result, one case of our study was probably associated with the history of stroke but some time delay between the events, so that it was not classified as clinically relevant leaflet thrombosis (also taken a RELM < 25% as diagnosed by MSCT into consideration). To conclude, there might be a need for a structured post TAVI imaging attempt, especially after bicuspid procedures. Mal-apposition, platelet activation, and high annular calcium amounts in BAV might impact sinus blood flow conditions and, therefore, the priming of HALT. Endocarditis is another reversible mechanism of non-SVD in the development of valve failure, described with an average of 5% in nearly all studies and a climax in the first two years [17], unfortunately still combined with high mortality. Endocarditis in this study was a rare event with only 0.9% reflecting one case that likewise led to one-year death but might be underestimated compared to current literature.

### Hemodynamic valve deterioration and bioprosthetic valve failure

HVD may be caused by SVD, valve thrombosis, or endocarditis. Therefore, an in-depth assessment of valve leaflet morphology and structure is crucial to differentiate between the given entity. We could identify early HVD in 16.8% of patients, distributed on 11.2% stage 1 and 5.6% stage 2 HVD according to the currently recommended classification [4]. Again, approximately 11% of cases were already documented during 30-day FU, which must be considered as high compared to current knowledge but seem to be an effect of the new streamlined algorithm that also includes morphological changes (HVD stage 1). However, nearly 4% of stage 2 HVD (i.e., non-PPM caused relevant gradient elevation) during the 30-day course is an issue to address and to further investigate in TAVI-treated bicuspid patients. As bioprosthetic valve failure (BVF) should be reported separately from subclinical BVD to adequately address the clinical relevance, we documented two events of BVF (1.9%), one stage 1 BVF with worsening of symptoms due to SVD-triggered HVD stage 2 and one stage 3 BVF (valve-related death) due to endocarditis of the bio-prosthesis. Compared to the current literature and the remarks above, the amount of BVF seems to be again higher in short-to-mid-term FU. However, the new classification according to VARC-3 was never addressed so far.

### Conclusion

There is critical evidence of early BVD after TAVI in BAV during one-year FU in one-third of patients, also lowering device success. The most frequently observed bioprosthetic valve dysfunction was NSVD due to patient prosthesis mismatch following TAVI with a balloon-expandable valve. Identifying predictors of BVD may help to optimize long-term valve performance in the future.

### Limitations

This study is a dual-center, retrospective analysis with associated unavoidable limitations due to its design. The frequencies of HALT/RELM must be handled with caution as cardiac CT wasn’t performed regularly. However, taking the low percentage of bicuspid valves in western countries into account, we provided first insights into the prevalence of BVD according to the new VARC-3 criteria in a bicuspid cohort covering more than a hundred patients.

## Supplementary Information

Below is the link to the electronic supplementary material.

Supplementary file1 (DOCX 30 KB)

## Abbreviations

BVD: Bioprosthetic dysfunction
BVF: Bioprosthetic valve failure
HALT: Hypo-attenuating leaflet thickening
HVD: Hemodynamic valve deterioration
LVOT: Left ventricular outflow tract
NSVD: Non-structural valve dysfunction
MSCT: Multislice computer tomography
PPI: Permanent pacemaker implantation
PPM: Permanent pacemaker implantation
PPI: Patient prosthesis mismatch
SVD: Structural valve deterioration
