# Supplementary material for: Bioprosthetic valve dysfunction and failure after TAVI in bicuspid aortic valve stenosis during one-year follow-up according to VARC-3
Source: Clin Res Cardiol. 2022 Jun 29;111(12):1358–66. doi: 10.1007/s00392-022-02052-9 (PMC9681687; doi:10.1007/s00392-022-02052-9)
Supplement: Supplementary file 1 — Supplementary file1 (DOCX 30 KB) [file 392_2022_2052_MOESM1_ESM.docx]

**SUPPLEMENTARY MATERIAL**

**Methods: VARC-3 Definitions of Bioprosthetic Valve Dysfunction (BVD)** (4)

Bioprosthetic valve dysfunction might be related to several etiologies like

- Structural Valve Deterioration (SVD, irreversible intrinsic changes to structural elements of the valve itself)
- Non-structural Valve Dysfunction (NSVD, including paravalvular leakage and prosthesis-patient mismatch)
- Endocarditis
- Thrombosis (Hypoattenuating leaflet thickening-HALT; reduced leaflet motion-RELM)

The stages of Hemodynamic Valve Deterioration (HVD) are defined by VARC-3 according to the progress (stage 1 up to 3). HVD may be caused by SVD but also by valve thrombosis or endocarditis. Bioprosthetic valve failure (BVF) is defined as consequence of BVD such as SVD-related Stage 3 hemodynamic valve deterioration and irreversible changes in hemodynamics, as well as clinical symptoms, including valve-related death and re-intervention.

**Table S1. Patient Clinical and Functional Characteristics (Overall population)**

| **Clinical data** | **Overall**  **(n=109; 100%)** | **No BVD**  **(n=75; 68.8%)** | **BVD**  **(n=34; 31.2%)** | **p-value** |
| --- | --- | --- | --- | --- |
| Age, years | 74.2 ± 7.7 | 74.3 ± 8.3 | 73.9 ± 6.4 | 0.779 |
| Gender, male | 75 (68.8) | 48 (64.0) | 29 (79.4) | 0.108 |
| BMI | 26.4 ± 4.7 | 26.1 ± 5.3 | 27.1 ± 6.5 | 0.415 |
| CAD | 43 (39.5) | 28 (37.3) | 15 (44.1) | 0.502 |
| Previous PCI | 17 (15.6) | 11 (14.7) | 6 (17.7) | 0.691 |
| Previous CABG | 6 (5.5) | 4 (5.3) | 2 (5.9) | 0.907 |
| Previous valve (non-aortic) | 2 (1.8) | 2 (2.7) | 0 (0.0) | 0.337 |
| Previous PPI | 12 (11.0) | 8 (10.7) | 4 (11.8) | 0.865 |
| Arterial hypertension | 73 (67.0) | 51 (68.0) | 22 (64.7) | 0.735 |
| PHT | 44 (40.4) | 32 (42.7) | 12 (35.3) | 0.467 |
| Diabetes mellitus | 15 (13.8) | 10 (13.3) | 5 (14.7) | 0.847 |
| PAD | 15 (13.8) | 12 (16.0) | 3 (8.8) | 0.314 |
| Porcelain aorta | 8 (7.3) | 7 (9.3) | 1 (2.9) | 0.236 |
| Previous RRT | 4 (3.7) | 4 (5.3) | 0 (0.0) | 0.170 |
| COPD | 21 (19.3) | 16 (21.3) | 5 (14.7) | 0.626 |
| Atrial fibrillation/flutter | 29 (26.6) | 18 (24.0) | 11 (32.4) | 0.361 |
| **Functional data** |  |  |  |  |
| STS, % | 2.5 ± 2.4 | 2.7 ± 2.7 | 2.0 ± 1.2 | 0.159 |
| LVEF, % | 52.3 ± 12.0 | 54.0 ± 10.9 | 48.4 ± 13.5 | *0.023** |
| AVA, cm^2^ | 0.71 ± 0.2 | 0.75 ± 0.2 | 0.64 ± 0.2 | *0.007** |
| dPmean, mmHg | 47.0 ± 15.8 | 45.8 ± 16.4 | 49.8 ± 14.1 | 0.223 |
| **MSCT data** |  |  |  |  |
| Bicuspid Type 0 | 4 (3.7) | 3 (4.0) | 1 (2.9) | 0.785 |
| Bicuspid Type 1 | 105 (96.3) | 72 (96.0) | 33 (97.1) | 0.785 |
| R/L | 91 (83.5) | 62 (82.7) | 29 (85.3) | 0.732 |
| L/N | 1 (0.9) | 1 (1.3) | 0 (0.0) | 0.499 |
| N/R | 13 (11.9) | 9 (12.0) | 4 (11.8) | 0.972 |
| AOA mean diameter, mm | 37.1 ± 4.3 | 36.8 ± 4.0 | 37.6 ± 5.0 | 0.378 |
| STJ mean diameter, mm | 31.8 ± 3.6 | 31.5 ± 3.6 | 32.4 ± 3.8 | 0.237 |
| SOV mean diameter, mm | 35.2 ± 4.0 | 35.0 ± 4.1 | 35.6 ± 3.6 | 0.499 |
| LVOT mean diameter, mm | 25.8 ± 3.3 | 25.5 ± 3.5 | 26.5 ± 2.8 | 0.161 |
| AN perimeter-derived diameter, mm | 26.5 ± 2.8 | 26.3 ± 3.0 | 27.8 ± 2.3 | 0.442 |
| AN perimeter, mm | 83.0 ± 8.7 | 82.6 ± 9.4 | 84.1 ± 7.1 | 0.391 |
| SAN ICD, mm | 25.7 ± 3.6 | 25.1 ± 3.6 | 17.0 ± 3.1 | *0.007** |
| SAN perimeter, mm | 85.5 ± 14.5 | 83.3 ± 14.5 | 90.5 ± 13.3 | *0.016** |
| Prosthesis-AN ratio  (>1=oversizing; <1=undersizing) | 1.1 ± 0.1 | 1.1 ± 0.1 | 1.1 ± 0.1 | 0.860 |
| Prosthesis-ICD ratio | 1.2 ± 0.1 | 1.2 ± 0.2 | 1.1 ± 0.2 | *0.032** |
| ICD-ratio | 1.0 ± 0.1 | 1.0 ± 0.1 | 1.0 ± 0.1 | *0.007** |
| TUBE (0.9-1.1) | 63 (57.8) | 45 (60.0) | 18 (52.9) | 0.489 |
| FLARE (>1.1) | 17 (15.6) | 8 (10.7) | 9 (26.5) | *0.035** |
| TAPER (<0.9) | 29 (26.6) | 22 (29.3) | 7 (20.6) | 0.338 |
| Height Raphe >50% | 43 (41.0) | 26 (36.1) | 17 (51.5) | 0.136 |
| Aortic Root Angulation (°) | 54.7 ± 10.7 | 53.6 ± 9.9 | 57.1 ± 12.0 | 0.104 |
| AVC, AU (total) | 4.100  [1.978-7.516] | 3.780  [1.754-6.876] | 4.984  [2.325-8.130] | 0.674 |
| AVC, AU (NCC) | 1.907  [1.029-3.516] | 1.901  [828-3.052] | 2.186  [1.200-3.965] | 0.291 |
| AVC, AU (RCC) | 1.144  [382-2.068] | 800  [358-2.472] | 1.272  [728-1.753] | 0.889 |
| AVC, AU (LCC) | 686  [245-1.888] | 583  [218-1.676] | 1.129  [344-2.073] | 0.983 |
| AVC, grading (total) |  |  |  |  |
| mild | 10 (9.2) | 7 (9.3) | 3 (8.8) | 0.932 |
| moderate | 9 (8.3) | 6 (8.0) | 3 (8.8) | 0.968 |
| severe | 90 (82.6) | 62 (82.7) | 28 (82.4) | 0.468 |
| LVOT-Calcification | 33 (44.0) | 20 (54.1) | 13 (38.2) | 0.572 |
| Values are mean ± SD, median ± interquartile range or n (%). *p-value < 0.05  AF=atrial fibrillation; AN=annulus; AOA=ascending aorta; AVA=aortic valve area; AVC=aortic valve calcification; BMI=body mass index; CABG=coronary artery bypass graft; CAD=coronary artery disease; CI=cardiac index; COPD=chronic obstructive pulmonary disease; CVD=cerebrovascular disease; dPmean/max=mean/max. transvalvular gradient; LVEF=Left ventricular ejection fraction; LVOT=Left ventricular outflow tract; PCI=percutaneous coronary intervention; PHT=pulmonary hypertension; PAD=peripheral artery disease; PPI=permanent pacemaker implantation; RRT=renal replacement therapy; SAN=supraannular; SOV=sinus of valsalva; STJ=sinutubular junction; | | | | |
